# Comparative efficacy and safety of olezarsen versus volanesorsen for familial chylomicronemia syndrome: a matching-adjusted indirect comparison

**DOI:** 10.57264/cer-2026-0069

**Published:** 2026-06-19

**Authors:** Handrean Soran, Asia Sikora Kessler, Montserrat Vera-Llonch, Veronica J Alexander, Paul Serafini, Divya Pushkarna, Elisabete Rodrigues, Erik Stroes, Ulrike Schatz, Ursula Kassner, Zalmai Hakimi, Aletta Falk, Koo Wilson

**Affiliations:** 1 NIHR/Welcome Trust Clinical Research Facility & Manchester University Hospital NHS Foundation Trust, Manchester, UK; 2 Ionis Pharmaceuticals Inc., Carlsbad, CA, USA; 3 Evidinno Outcomes Research Inc., Vancouver, BC, Canada; 4 Department of Endocrinology, ULS Sao Joao, Faculty of Medicine, University of Porto (FMUP), Porto, Portugal; 5 Department of Vascular Medicine, Amsterdam University Medical Center, Amsterdam, The Netherlands; 6 Department of Internal Medicine III of the University Hospital Dresden, Technical University of Dresden, Germany; 7 Department of Endocrinology, Division of Lipid Disorders, Berlin, Germany; 8 Sobi, Stockholm, Sweden

**Keywords:** familial chylomicronemia syndrome, hypertriglyceridemia, indirect treatment comparison, olezarsen, volanesorsen

## Abstract

**Background:**

Familial chylomicronemia syndrome (FCS) is a rare genetic disorder characterized by severe hypertriglyceridemia causing recurrent acute pancreatitis (AP). Since FCS is a genetic deficiency in functional lipoprotein lipase, conventional triglyceride-lowering therapies are ineffective in this metabolic disorder. Apolipoprotein C-III (apoC-III) inhibitors, including volanesorsen and olezarsen, have emerged as targeted treatments for FCS.

**Aim:**

To compare the efficacy and safety of olezarsen 80 mg every four weeks (Q4W) versus volanesorsen 300 mg weekly (QW) in patients with FCS using an anchored matching-adjusted indirect comparison.

**Materials & methods:**

Individual patient data from the Balance trial of olezarsen (n = 45) were weighted to match baseline characteristics reported in the APPROACH trial of volanesorsen (n = 66). Outcomes included percent change in fasting triglycerides (TG) and apoC-III at 26 and 52 weeks, and risks of AP events and adverse events at 52 weeks.

**Results:**

At 52 weeks, mean differences in fasting TG and apoC-III were -27.4% (95% CI: -69.4, 14.5) and -21.3% (95% CI: -61.9, 19.3). Relative risks of AP, treatment-emergent adverse events, and serious adverse events were 0.23 (95% CI: 0.01, 5.02), 0.87 (95% CI: 0.66, 1.13) and 0.50 (95% CI: 0.08, 3.26) at week 52. The rate ratio for AP events per patient-year was 0.06 (95% CI: 0.003, 1.41). No comparisons were statistically significant.

**Conclusion:**

In this matching-adjusted indirect comparison, no statistically significant differences in outcomes were observed between olezarsen and volanesorsen in patients with FCS. These findings provide important comparative context in a setting where head-to-head evidence is unavailable.

## Plain language summary

**What is this article about?:**

Familial chylomicronemia syndrome (FCS) is a rare inherited condition where the body cannot properly break down specific type of fats in the blood, called triglycerides. This leads to very high triglyceride levels and can cause repeated episodes of acute pancreatitis (AP) (a painful and serious inflammation of the pancreas). Conventional triglyceride-lowering therapies usually do not sufficiently reduce triglycerides for people with FCS, so newer drugs that target a protein called apolipoprotein C-III (apoC-III; like olezarsen and volanesorsen) have been approved to treat FCS. The study compared the newer treatment olezarsen (administered at 80 mg once per four weeks) with volanesorsen (administered at 300 mg once per week) to see how well they work and how safe they are for people with FCS.

**How was the study was done?:**

Individual patient data from a clinical trial of olezarsen was adjusted to make the patient profile comparable to that of a clinical trial of volanesorsen (the comparator of interest) based on age, sex, race/ethnicity, body mass index, triglyceride levels and history of AP. Researchers then assessed how much olezarsen decreased triglyceride and apoC-III levels compared with volanesorsen, as well as how likely patients receiving olezarsen were to experience AP and adverse events compared with patients receiving volanesorsen.

**What were the results?:**

After 1 year of treatment, patients who received olezarsen reduced their triglyceride and apoC-III levels by 27% more and 21% more, respectively, compared with those who received volanesorsen. They also were 77% less likely to experience AP and 13% less likely to experience an adverse event. However, because the olezarsen and volanesorsen studies were both small, these differences were not large enough to conclude that one drug is more effective or safe.

**What do the results mean?:**

In this study, there was no evidence of differences in outcomes between olezarsen and volanesorsen in the treatment of patients with FCS. This is a first comparison of two drugs which have not been compared directly in a randomized controlled clinical trial.

Familial chylomicronemia syndrome (FCS) is a rare genetic disorder that causes primary hypertriglyceridemia [1]. Its prevalence in the general population is estimated to range from 1 to 13 cases per million individuals [2,3]. Clinically, FCS presents with recurrent episodes of acute pancreatitis (AP), eruptive xanthomas, lipemia retinalis, hepatosplenomegaly, cognitive symptoms and abdominal pain, leading to significant morbidity and decreased quality of life. Standard lipid-lowering therapies, such as fibrates, omega-3 fatty acids, and statins are largely ineffective in FCS due to their reliance on lipoprotein lipase (LPL) activity [4]. Biallelic loss-of-function mutations abolish LPL activity in FCS, rendering these therapies unable to reduce triglyceride (TG) levels meaningfully [5]. Additionally, while lipoprotein apheresis has been used as an adjunct to lower triglycerides in select patients with familial hypercholesterolemia, its uptake is impacted by the need for biweekly, in-center administration and by limited availability [6]. This highlights an unmet therapeutic need in this patient population.

Advances in molecular therapeutics have shifted the paradigm of FCS management toward ribonucleic acid (RNA) -targeted drugs aimed at modulating apolipoprotein C-III (apoC-III), a key regulator of TG metabolism that inhibits LPL activity and is also thought to inhibit LPL-independent TG lipolysis through hepatic lipase [7,8]. Volanesorsen (Waylivra^®^) 285 mg weekly or biweekly, a second-generation antisense oligonucleotide targeting apoC-III messenger RNA, was the first agent approved for genetically confirmed FCS by the EMA in 2019 based on its efficacy in reducing TG levels by over 70% in pivotal trials [9]. However, its clinical use has been constrained by notable safety concerns, particularly thrombocytopenia, necessitating frequent laboratory monitoring [10,11]. As a consequence, the US FDA declined to approve volanesorsen for FCS.

Olezarsen (Tryngolza^®^), a second-generation N-acetyl galactosamine-conjugated antisense oligonucleotide also targeting apoC-III, offers enhanced targeting of the antisense oligonucleotide to the hepatocyte and reduced systemic exposure, translating to decreased injection volume, reduced dosing frequency [12], and potentially improved safety and tolerability compared with volanesorsen. Olezarsen 80 mg once a month (Q4W) recently received Committee for Medicinal Products for Human Use approval from the EMA as an adjunct to diet in adult patients for the treatment of genetically confirmed FCS [13], and was the first agent approved by the FDA in 2024 as an adjunct to low-fat diet in the management of FCS [14]. In the Phase III Balance trial, olezarsen achieved robust 6-month triglyceride reductions (-43.5% in the 80 mg arm relative to placebo) and reductions in the rate of AP events (88% reduction relative to placebo) with favorable safety results [15]. Consistent with olezarsen’s favorable safety profile, the US Prescribing Information and EMA Summary of Product Characteristics (SmPC) labels do not impose platelet, liver or kidney monitoring requirements on olezarsen, whereas the volanesorsen EU SmPC [16] mandates regular platelet monitoring and dose-adjustment algorithms to mitigate thrombocytopenia risk. Additionally, another apoC-III inhibitor, plozasiran, has received FDA approval [14] at a dose of 25 mg every 3 months and recently received EMA marketing authorization. This further expands the therapeutic landscape for FCS, offering an alternative dosing strategy.

Due to the absence of head-to-head trials comparing these agents directly, matching-adjusted indirect comparisons (MAICs) represent a useful and widely accepted method to evaluate their relative efficacy and safety which are endorsed by health technology assessment agencies such as the National Institute for Health and Care Excellence (NICE) [17]. This methodology adjusts for differences in baseline characteristics using individual patient-level data (IPD) from one trial and aggregate data from another, thereby improving the comparability of outcomes across studies.

The aim of this study was to conduct an MAIC between olezarsen 80 mg Q4W and volanesorsen 300 mg every week (QW) in patients with FCS, with the objective of estimating their comparative efficacy and safety. This may provide important comparative evidence in the absence of head-to-head trials.

## Materials & methods

### Data sources

A systematic literature review was performed following the Cochrane methodology to ensure that all relevant studies were captured [18]. Eligible studies were identified by searching Embase, MEDLINE^®^ and CENTRAL via OvidSP. Grey literature searches of relevant conferences from 2022 to 2024 and US and European clinical trial registries were also conducted. The PICO criteria and search strategies are reported in Appendix A.

**Figure 1.**
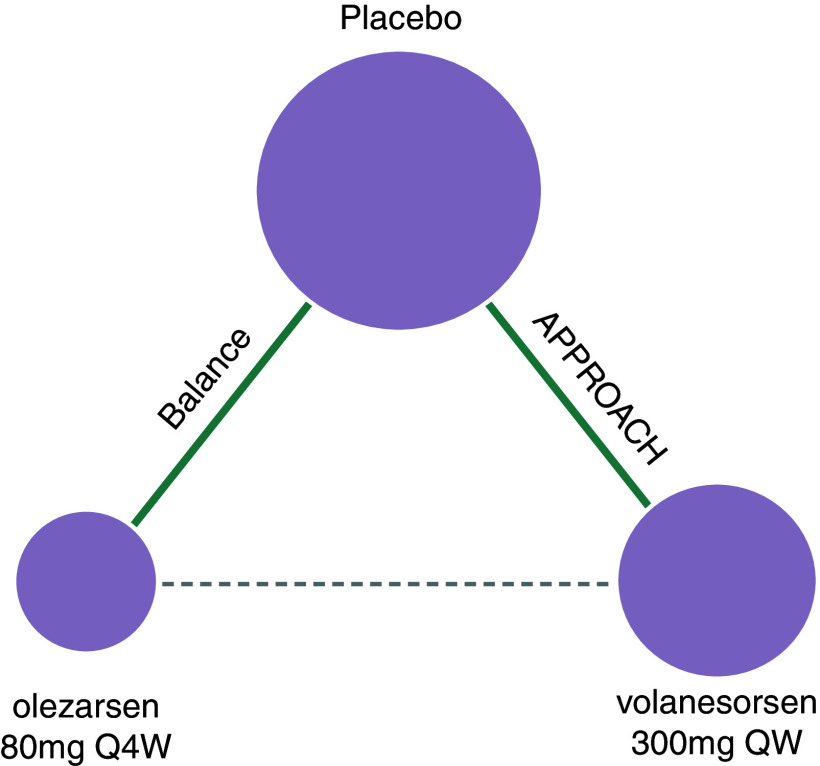
Network diagram for the comparison of olezarsen 80 mg Q4W to volanesorsen 300 mg QW. QW: Weekly; Q4W: Every 4 weeks.

Three trials met the criteria for inclusion in the MAIC: one olezarsen study (Balance [15]) and two volanesorsen studies (APPROACH [19] and COMPASS [20]). The Balance [15] and APPROACH [19] trials were selected for inclusion in the MAIC (Figure 1). The APPROACH [19] trial was selected over COMPASS [20] because it served as the pivotal study evaluating volanesorsen in patients with FCS, whereas COMPASS [20] included individuals with severe hypertriglyceridemia, with FCS patients constituting only a minor subgroup. Both the Balance and APPROACH trials were judged to be of ‘low concern’ for bias according to the Cochrane risk of bias assessment tool for clinical trials. The inclusion of only a single olezarsen trial consisting of 45 patients represented a limitation of the MAIC.

### Weighting & matching procedure

Patients in the Balance [15] trial were assigned weights through a logistic regression approach to match the distribution of effect-modifiers observed in the APPROACH [19] trial. Matching was conducted separately for each end point and timepoint. Continuous variables were matched on mean values only, not variances, to preserve effective sample size (ESS) and statistical power due to the rarity of FCS and therefore small trial patient numbers. Post-matching, the reweighted IPD from Balance [15] was used to generate adjusted aggregate-level estimates for olezarsen. These were compared with the published outcomes for volanesorsen using a Bucher indirect treatment comparison (ITC) method, anchored through the respective placebo arms. Sandwich estimators were used to calculate standard errors of the weighted outcomes [21].

### Outcomes of interest

Continuous outcomes of interest included percent improvement in fasting TG and apoC-III at 26 and 52 weeks. Dichotomous outcomes of interest included the risk of at least one AP event, risk of at least one treatment-emergent adverse event (TEAE), risk of at least one related TEAE, risk of at least one mild related TEAE and risk of at least one serious adverse event (SAE) at 52 weeks. In addition to the risk of at least one AP event, the number of AP events per patient-year of follow-up were compared. Proximate timepoints were used where necessary to enable analyses.

### Selection of matching variables

The selection of baseline variables for matching focused on identifying treatment effect-modifiers [17], which are variables that influence the relative treatment effect between comparators. The following variables were assessed: age, sex (female), race/ethnicity (White), body mass index (BMI), baseline fasting TG, number of prior AP events, and history of at least one AP event in the past 5 years. Treatment-effect modifier status was assessed for each end point separately, per NICE’s recommendation [17]. This approach is preferred because imbalance in a variable will only bias the indirect estimate if that variable is a treatment effect modifier, treatment effect modifier status can vary by outcome, and adjusting for more variables can decrease the ESS and therefore the power of the analysis.

Baseline fasting TG was classified *a priori* as a treatment effect-modifier for all outcomes based on clinical consensus. For other variables, treatment-by-covariate interaction terms were assessed based on IPD from Balance [15], using linear regression for continuous outcomes (to test for association with the mean difference [MD]), binomial regression for dichotomous outcomes (to test for association with the relative risk) and Poisson regression for count outcomes (to test for association with the rate ratio). A variable was considered a treatment effect-modifier if the interaction met either statistical significance (p < 0.05) or exploratory significance (0.05 ≤ p < 0.20). Although the use of a liberal, exploratory threshold for significance may inflate the probability of adjusting for a variable that is not a treatment effect modifier, this risk was considered acceptable because the inclusion of a variable which is not an effect modifier will not bias the indirect comparison, although it may reduce power and precision. Identified effect-modifiers were subsequently included in the weighting model for each respective outcome.

### Placebo-anchored MAIC

The placebo-anchored MAIC [17] was conducted using IPD from the Balance [15] study and aggregate-level data from the APPROACH [19] trial. For each outcome, Balance [15] participants were assigned patient-level weights using logistic regression to match the summary baseline treatment effect modifiers reported in APPROACH [19].

Matching was conducted on mean values of continuous covariates only (not variances). Due to the small sizes of the available trials in this rare disease population, matching on higher-order moments (e.g., variances) may have increased weight variability and caused the estimates to depend on a small number of individuals, leading to unstable estimates and loss of statistical power and precision. Based on this consideration, matching was conducted on the first-order moments (i.e., means) only. This decision may allow some residual imbalance in the distribution of continuous covariates.

In Balance, for the 26-week analysis timepoint (calculated as the mean of 23, 25 and 27 weeks), when a component measurement was missing for a patient, it was calculated as the mean of the available timepoints. This occurred for apoC-III (two patients were missing one measurement each, one at week 23 and the other at week 27). No missing data were present for any other timepoint or outcome.

The reweighted Balance [15] data were used to estimate adjusted treatment effects for olezarsen relative to placebo, using a sandwich estimator to compute weighted standard errors. The adjusted treatment effects from Balance [15] were then combined with aggregate-level treatment effects from APPROACH [19] to indirectly compare olezarsen to volanesorsen. For continuous outcomes, the treatment effect was estimated as the MD in percent change from baseline. For dichotomous outcomes, risk ratios were calculated, and for count outcomes, rate ratios were used. The primary assumption of this methodology is conditional constancy of relative effects [17], meaning that all treatment effect modifiers that differ between populations are measured and conditioned on, such that treatment effects are the same conditional on these covariates.

Sensitivity analyses included:

- Unmatched Bucher ITC [22] conducted using unadjusted estimates to assess the impact of matching on the results.
- MAIC with full covariate matching: Included all baseline variables regardless of statistical significance of their interaction terms, to assess the sensitivity of the results to the treatment effect modifier selection procedure.

## Results

### Evidence base

The characteristics of the Balance and APPROACH studies are summarized in Table 1. Both studies were Phase III international randomized controlled trials, however Balance required a higher baseline TG for inclusion and, correspondingly, had a higher mean baseline TG.

**Table 1.** Summary of the characteristics of the included studies.

Outcome	Balance [15]	APPROACH [19]
Data source	IPD	Aggregate published data
Sample size	45^†^	66
Active therapy	Olezarsen 80 mg Q4W	Volanesorsen 300 mg QW
Phase	III	III
Blinding	Double	Double
Region	International	International
Minimum TG threshold	880 mg/dl	750 mg/dl
Mean Baseline TG	2630 mg/dl	2209 mg/dl

† Restricted to patients included in the statistical analyses (i.e., excluding 50 mg olezarsen).

IPD: Individual patient data; Q4W: Every 4 weeks; QW: Once weekly; TG: Triglyceride.

### Selection of matching variables

For each outcome of interest, the p-value for the interaction term between each candidate effect modifier (other than baseline fasting TG) and treatment arm is presented in Table 2. For the percent change in fasting TG and apoC-III outcomes, age, White race/ethnicity, and 5-year AP history were identified as additional effect-modifiers. For the number of AP events per patient-year, body mass index (BMI) was identified as an additional effect-modifier. For the risk of at least one SAE, 5-year AP history was identified as an additional effect-modifier. No additional effect-modifiers were identified for the AP risk, TEAE risk or related TEAE risk outcomes.

**Table 2.** Selection of matching variables.

Outcome	p-value for the interaction term				
	Age	Sex	White race	BMI	5-year AP history
Percent change in fasting TG	0.14^†^	0.91	0.008^‡^	0.91	0.01^‡^
Percent change in fasting apoC-III	0.006^‡^	0.32	0.06^†^	0.62	0.01^‡^
AP, ≥1 event	0.30	1.00	0.99	0.30	1.00
AP, events, n	0.23	N/E	0.57	0.13^†^	N/E
TEAE, any	0.37	0.99	1.00	0.36	0.99
TRAE, any	0.24	0.60	0.99	0.65	0.58
TRAE, mild	0.31	0.99	0.99	0.31	0.45
SAE, any	0.95	0.34	0.99	0.97	0.15^†^

Columns age through 10-year AP history contain the p-value for the statistical test of an interaction between the variable corresponding to that column and treatment arm on the outcome in that row. Variables identified as treatment effect modifiers for each outcome are marked with a superscript symbol. Baseline fasting TG was included as an effect modifier for all outcomes a priori based on clinical considerations, and therefore the interaction term was not tested for that variable.

† Exploratory significance (0.20 > p ≥ 0.05).

‡ Statistical significance (p < 0.05).

AP: Acute pancreatitis; apoB-48: Apolipoprotein B-48; apoC-III: Apolipoprotein C-III; BMI: Body mass index; HDL-C: High-density lipoprotein cholesterol; N/E: Not estimable; SAE: Serious adverse event; TEAE: Treatment-emergent adverse event; TG: Triglyceride; TRAE: Treatment-related adverse event.

### Matching procedure

The eligible population from Balance [15] included 22 patients treated with olezarsen 80 mg Q4W and 23 patients who received placebo. In the APPROACH [19] study, the corresponding eligible cohort comprised 33 patients treated with volanesorsen 300 mg QW and 33 patients who received placebo. The baseline characteristics for APPROACH [19] before matching, and Balance [15] after matching, are presented for the experimental arms in Table 3 and in the placebo arms in Table 4. Following the matching procedure, the distributions of covariates used for matching were closely aligned between the APPROACH [19] and Balance [15] study populations, indicating successful balance across matched variables.

**Table 3.** Baseline characteristics in the experimental arms of APPROACH (volanesorsen 300 mg QW) and Balance (olezarsen 80 mg Q4W) before and after matching.

Characteristic	APPROACH [19] (Volanesorsen 300 mg QW)	Balance [15] (Olezarsen 80 mg Q4W)				
		MAIC analysis (By outcome set)				
	Published	Unweighted	# of AP events	apoC-III and TG	SAEs	AP, TEAE and TRAE risk
Sample size (N)	33	22	20.7^‡^	19.2^‡^	19.2^‡^	20.8^‡^
Age (years)	47.0	47.7	47.1	**47.0** ^†^	48.6	47.7
Female sex	52%	50%	48%	48%	46%	50%
White race	73%	77%	82%	**73%** ^†^	85%	82%
BMI (km/m^2^)	25.9	25.1	**25.9** ^†^	25.2	25.4	25.4
Fasting TG (mg/dl)	2267	2613.1	**2267** ^†^	**2267** ^†^	**2267** ^†^	**2267** ^†^
5-year AP history, ≥1 events	73%	68%	67%	**73%** ^†^	58%	67%
5-year AP history, events, n	0.9	1.7	1.8	1.9	**0.9** ^†^	1.8

Within each outcome set, variables that were matched to APPROACH are bolded.

† Matching was conducted on this outcome set (these cells are bolded).

‡ Value is ESS as opposed to sample size.

AP: Acute pancreatitis; Apo: Apolipoprotein; BMI: Body mass index; ESS: Effective sample size; HDL-C: High-density lipoprotein cholesterol; SAE: Serious adverse event; TEAE: Treatment-emergent adverse event; TRAE: Treatment-related adverse event; TG: Triglyceride.

**Table 4.** Baseline characteristics in the placebo arms of APPROACH and Balance before and after matching.

Characteristic	APPROACH [19] (Placebo)	Balance [15] (Placebo)				
		MAIC analysis (By outcome set)				
	Published	Unweighted	# of AP events	apoC-III and TG	SAEs	AP, TEAE and TRAE risk
Sample size	33	23	19.9^‡^	16.0^‡^	14.4^‡^	20.2^‡^
Age (years)	46.0	44.0	43.1	**46.0** ^†^	45.0	42.9
Female sex	58%	52%	50%	58%	52%	53%
White race	88%	96%	95%	**88%** ^†^	98%	95%
BMI (km/m^2^)	24.1	24.2	**24.1** ^†^	24.0	23.1	23.6
Fasting TG (mg/dl)	2152.0	2595.7	**2152.0** ^†^	**2152.0** ^†^	**2152.1** ^†^	**2152.0** ^†^
5-year AP history, ≥1 events	79%	61%	63%	**79%** ^†^	35%	62%
5-year AP history, events, n	0.7	3.8	3.5	4.1	**0.7** ^†^	3.4

Within each outcome set, variables that were matched to APPROACH are bolded.

† Matching was conducted on this outcome set (these cells are bolded).

‡ Value is ESS as opposed to sample size.

AP: Acute pancreatitis; Apo: Apolipoprotein; BESS: Effective sample size; BMI: Body mass index; HDL-C: High-density lipoprotein cholesterol; SAE: Serious adverse event; TEAE: Treatment-emergent adverse event; TRAE: Treatment-related adverse event; TG: Triglyceride.

After matching, the ESSs of the olezarsen and placebo arms of Balance [15] were reduced by 6% and 17% for number of AP events, 13% and 30% for apoC-III and TG, 13% and 37% for SAEs, and 5% and 12% for the remaining outcomes (the risk of at least one AP episode, TEAE and related TEAE). Histograms of rescaled (mean of 1) patient weights are presented in Appendix B. The distributions showed little skew (the median ranged from 0.8 to 1.1) and there were no extreme outliers (the maximum rescaled patient weight across all analyses was 3.1). While the resulting reductions in ESS represent a limitation to precision, these reductions were smaller than the average of 45% reported in one literature review of MAICs [23].

### Primary placebo-anchored matching adjusted indirect comparison

#### Lipid & apolipoprotein parameters

**Figure 2.**
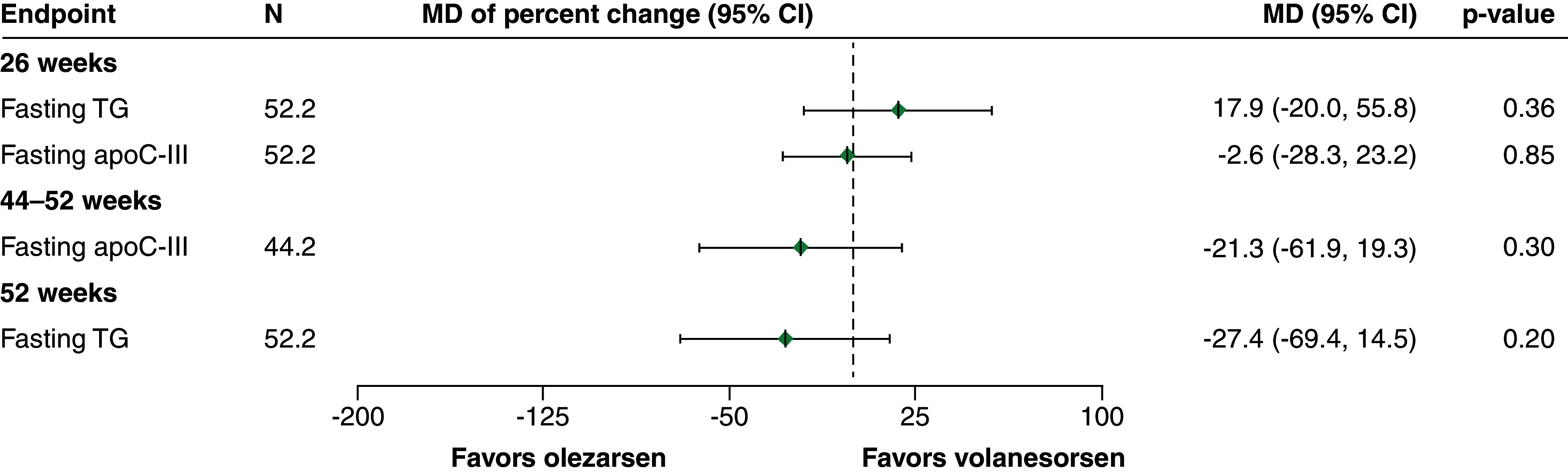
Results of placebo-anchored matching adjusted indirect comparisons of olezarsen versus volanesorsen on improvement in lipid and apolipoprotein parameters. Apo: Apolipoprotein; HDL-C: High-density lipoprotein cholesterol; MAIC: Matching adjusted indirect comparison; MD: Mean difference; N: Effective sample size in the olezarsen and volanesorsen arms; TG: Triglyceride.

At 26 weeks, MDs in percentage changes from baseline for fasting TG and apoC-III were 17.9% (95% CI: -20.0, 55.8) and -2.6% (95% CI: -28.3, 23.2), respectively (Figure 2). At 52 weeks, these MDs were -27.4% (95% CI: -69.4, 14.5) and -21.3% (95% CI: -61.9, 19.3). However, none of these differences reached statistical significance.

#### Acute pancreatitis (AP)

**Figure 3.**
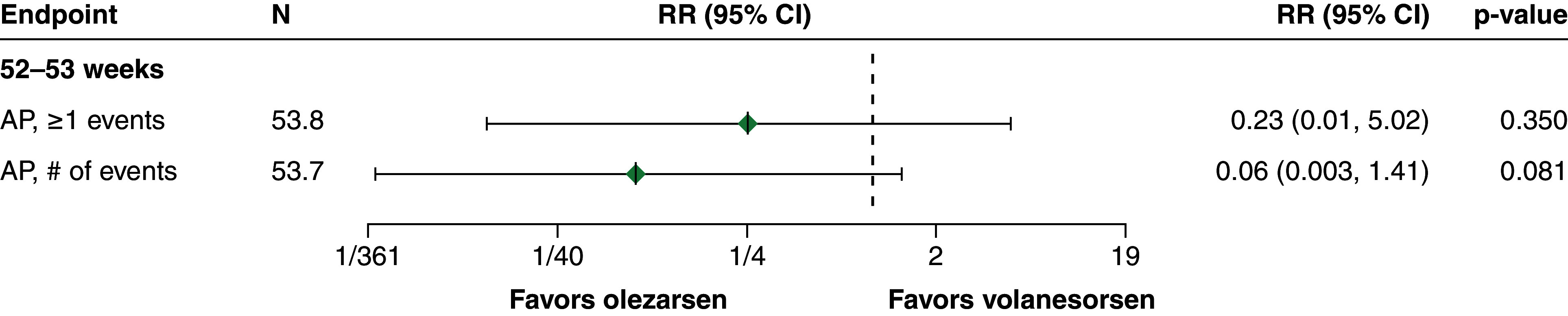
Results of placebo-anchored matching adjusted indirect comparison of olezarsen versus volanesorsen on risk of acute pancreatitis. AP: Acute pancreatitis; HDL-C: High-density lipoprotein cholesterol; MAIC: Matching adjusted indirect comparison; MD: Mean difference; N: Number of patients in the olezarsen and volanesorsen arms; RR: Risk ratio (‘≥1 events’) or Rate Ratio (‘# [number] of events’).

Over 52 weeks, the relative risk of ≥1 AP event was 0.23 (95% CI: 0.01, 5.02) and the rate ratio for the number of AP events per patient-year of follow-up was 0.06 (95% CI: 0.003, 1.41) (Figure 3). However, neither of these differences were statistically significant.

#### Safety

**Figure 4.**
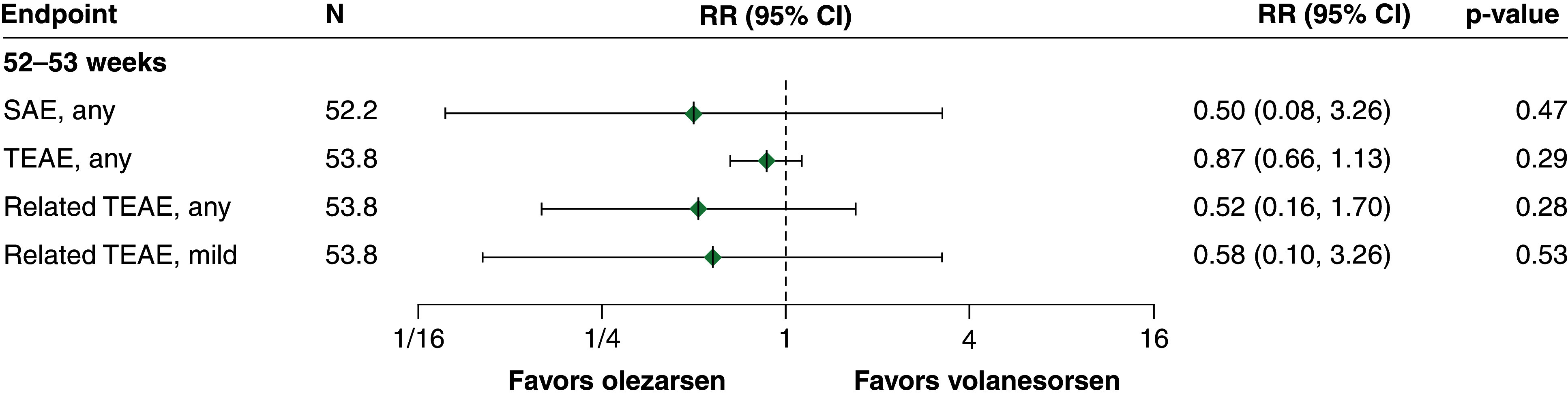
Results of placebo-anchored matching adjusted indirect comparison of olezarsen versus volanesorsen on risk of adverse events. AE: Adverse event; N: Number of patients in the olezarsen and volanesorsen arm; RR: Risk ratio; SAE: Serious AE; TEAE: Treatment-emergent AE.

Over 52 weeks, the relative risks of TEAEs, SAEs, related TEAEs and mild related TEAEs were 0.87 (95% CI: 0.66, 1.13), 0.50 (95% CI: 0.08, 3.26), 0.52 (95% CI: 0.16, 1.70) and 0.58 (95% CI: 0.10, 3.26), respectively (Figure 4). However, none of these differences reached statistical significance.

#### Sensitivity analyses

The results of the sensitivity analyses are compared with the primary analysis in Table 5. Matching did not impact study results based on statistical significance. When matching was conducted on all variables regardless of outcome, point estimates were generally similar to the primary analysis, however the relative risk of at least one SAE for olezarsen shifted from 0.5 to 0.18 and became statistically significant. Additionally, the reduction in the number of AP events per patient-year also became statistically significant despite a more modest change in point estimate from a rate ratio of 0.06 to 0.05.

**Table 5.** Comparison of primary and sensitivity matching adjusted indirect comparison results for the comparison of olezarsen 80 mg Q4W to volanesorsen 300 mg QW.

End point	Statistic	Week(s)	Estimate (95% CI)		
			Primary MAIC	Unmatched Bucher ITC	Sensitivity MAIC
Fasting TG	MD	26	17.9 (-20.02, 55.8)	32.8 (2.2, 63.5)^†^	17.1 (-21.7, 55.8)
		52	-27.4 (-69.4, 14.5)	-10.4 (-44.7, 23.9)	-28.6 (-71.4, 14.3)
Acute pancreatitis, ≥1 events	RR	52–53	0.23 (0.01, 5.02)	0.45 (0.02, 8.91)	0.21 (0.01, 4.54)
Acute pancreatitis, events, n	IRR	52–53	0.06 (0.003, 1.41)	0.13 (0.01, 2.51)	0.05 (0.002, 1.07)^†^
Fasting apoC-III	MD	26	-2.6 (-28.3, 23.2)	4.1 (-18.7, 26.9)	-2.7 (-29.2, 23.8)
		44–52	-21.3 (-61.9, 19.3)	-12.2 (-42.0, 17.7)	-21.2 (-62.2, 19.7)
SAE, any	RR	52–53	0.50 (0.08, 3.3)	0.25 (0.05, 1.19)	0.18 (0.03, 0.98)^†^
TEAE, any	RR	52–53	0.87 (0.66, 1.13)	0.88 (0.705, 1.085)	0.81 (0.62, 1.07)
TRAE, any	RR	52–53	0.52 (0.16, 1.70)	0.57 (0.19, 1.68)	0.61 (0.17, 2.18)
TRAE, mild	RR	52–53	0.58 (0.10, 3.26)	0.65 (0.13, 3.29)	0.90 (0.15, 5.54)

The unmatched results in this table may not exactly match the results of the Bucher indirect treatment comparison due to methodological differences and because a different imputation method was used in this analysis than in the Balance publication.

† Statistical significance (p < 0.05).

HDL-C: High-density lipoprotein cholesterol; IRR: Rate ratio; ITC: Indirect treatment comparison; MAIC: Matching adjusted indirect comparison; MD: Mean difference; RR: Risk ratio; SAE: Serious adverse event; TEAE: Treatment-emergent adverse event; TG: Triglyceride; TRAE: Treatment-related adverse event.

## Discussion

This study sought to generate comparative efficacy and safety evidence for olezarsen 80 mg Q4W versus volanesorsen 300 mg QW in FCS by conducting a placebo-anchored MAIC using IPD from Balance [15] (olezarsen) and published aggregate data from APPROACH [19] (volanesorsen).

There was no evidence of difference in either mid-term (26-week) efficacy or long-term (52-week) efficacy and safety between olezarsen and volanesorsen across key clinical end points. Although certain point estimates were close to the null, they were accompanied by wide confidence intervals and therefore should not be interpreted as evidence of equivalence. These indeterminate results reflect the small sample sizes of the Balance and APPROACH trials, the loss of ESS due to the weighting procedure, the variability of lipid parameters in this population, and the low event rates for AP and safety outcomes.

Certain nonsignificant trends across outcomes and timepoints were apparent among the point estimates. For example, whereas short-term fasting TG reduction numerically favored volanesorsen and short-term apoC-III reduction was similar for both therapies, both outcomes numerically favored olezarsen at 52 weeks. Additionally, rates of AP and safety (TEAE, SAE and related TEAE) events were lower for olezarsen. These numerical differences in the rates of adverse events may reflect the fact that rates of platelet count reductions (33% vs 0%) and injection site reactions (61% vs 14%) were much higher in the volanesorsen arm of APPROACH [19] than the olezarsen 80 mg arm of Balance [15]. However, these trends should not be overinterpreted due to the small, heavily modeled dataset, as well as the assessment of multiple outcomes, timepoints and analysis sets without multiplicity adjustment.

A recent network meta-analysis comparing apoC-III inhibitors (volanesorsen, olezarsen, plozasiran) across 10 RCTs comprising 1,244 patients concluded that volanesorsen 300 mg QW ranked highest for percent TG reduction, while overall AE rates did not differ significantly among agents; however, the timepoint(s) being pooled were unclear, and the authors cautioned that small samples and heterogeneity limit confidence in treatment rankings [24]. In parallel, an olezarsen-specific meta-analysis of four RCTs comprising 202 patients reported significant TG and apoC-III reductions without excessive overall AEs or SAEs versus placebo, again in relatively small cohorts [25]. Our MAIC, by weighting IPD from Balance [15] to APPROACH’s [19] baseline profile and anchoring via placebo, extends this literature by comparing olezarsen to volanesorsen in FCS, rather than across high TG populations, and by estimating relative risks for AP and AEs, outcomes rarely synthesized across agents.

Nevertheless, this analysis has some limitations. First, the precision of the results was reduced by the fact that only one olezarsen trial was identified, the loss of ESS in the Balance [15] trial after weighting and the small sizes of the Balance [15] and APPROACH [19] trials, because FCS is an ultra-rare condition. This was mitigated by adjusting for effect modifiers only, thereby minimizing bias without unnecessarily sacrificing precision. The inclusion of a single olezarsen study also made it impossible to test the stability of the results across MAICs using IPD from different trial populations, which limited the robustness of the analysis.

Second, many patients in the volanesorsen arm of APPROACH experienced dose pauses or switched to every-two-weeks (Q2W) administration in response to platelet count reductions, not because adequate efficacy had been achieved. This highlights how the safety profile of volanesorsen may directly compromise efficacy, as treatment interruptions and reduced dosing frequency can limit drug exposure and attenuate therapeutic effect. Conversely, there were no platelet count reductions in either olezarsen arm (50 mg or 80 mg) of Balance, and hence no platelet-count-related pauses. This may be reflected in the numerical differences in safety results, and it could also have reduced the efficacy in the volanesorsen group due to the less frequent administration schedule. However, the EMA label for volanesorsen indicates that patients can pause treatment or switch from QW to Q2W administration in response to platelet count reductions [16], and therefore this is consistent with real world use of volanesorsen.

Third, dietary factors were not adjusted for in this analysis. Differences in dietary adherence could represent an important source of residual heterogeneity, as both olezarsen and volanesorsen are indicated as adjuncts to a low-fat diet. However, this may have been mitigated by the fact that both trials incorporated a dietary run-in period requiring participants to consume no more than 20 g of fat per day (6 weeks in APPROACH and 2 weeks in Balance), and both trials provided ongoing dietary counseling.

Fourth, treatment effect modifiers were identified using a data-driven approach in which interactions were tested within the Balance IPD. While best practices, such as those outlined by the NICE Decision Support Unit, suggest that effect modifiers should ideally be identified *a priori* using either external quantitative evidence, clinical opinion, or systematic review, such evidence is currently limited in this disease area. To mitigate the risk of *post-hoc* bias, the selection criteria were strictly pre-specified prior to the analysis. Furthermore, a sensitivity analysis adjusting for all candidate variables regardless of statistical significance was conducted and yielded results consistent with the primary analysis, suggesting that the data-driven selection process did not fundamentally alter the conclusions or introduce significant instability into the model.

Fifth, some cross-trial differences were not accounted for in the MAIC, and may challenge the assumption that, after adjustment for observed effect modifiers, the relative treatment effects are transportable between trials. Whereas Balance [15] recruited patients with a fasting TG of at least 880 mg/dl and genetic confirmation of FCS, APPROACH [19] recruited patients with a fasting TG of at least 750 mg/dl and either genetic confirmation of FCS or low LPL activity. Consequently, patients in Balance may have had more severe disease, as reflected in the higher mean baseline fasting TG in Balance [15]. Baseline TG severity and differences in the underlying LPL-pathway defect may modify lipid responses and pancreatitis risk, as was found in subgroup analyses of the APPROACH trial [26], and therefore could impact the comparability of relative treatment effects across studies. Assessment schedules varied slightly between trials for long-term fasting apoC-III, necessitating proximate timepoint alignment (52 weeks for olezarsen and 44 weeks for volanesorsen). As a consequence, long-term reduction in apoC-III for olezarsen may have been greater because patients in Balance had longer drug exposure. Matching was restricted to summary-level means for continuous variables, meaning that differences in the dispersion or shape of covariate distributions between trials could not be accounted for, and this may contribute to greater residual confounding. These cross-trial differences, along with any unmeasured or unreported effect modifiers, may have influenced the estimates and should be considered when interpreting the findings. Overall, in rare disease populations with small sample sizes, MAIC analyses can have limitations and do not replace head-to-head trials.

## Conclusion

In this MAIC, there was no evidence of differences in outcomes between olezarsen and volanesorsen in the treatment of patients with FCS. In the absence of statistically significant evidence of a difference in efficacy, treatment decisions should remain individualized and consider factors beyond comparative efficacy. Where clinically appropriate, switching to olezarsen may reduce patient burden due to its less frequent injection schedule and because it does not require platelet monitoring. Olezarsen’s generally mild and transient impacts on liver function test parameters may also be a relevant consideration for some patients. These findings provide important comparative context in a setting where head-to-head evidence is unavailable.

## Summary points

- Familial chylomicronemia syndrome (FCS) is a rare genetic disorder characterized by severe hypertriglyceridemia and recurrent acute pancreatitis.
- Apolipoprotein C-III inhibitors, including olezarsen and volanesorsen, have emerged as targeted treatment options for FCS.
- This study used a matching-adjusted indirect comparison to compare olezarsen 80 mg every 4 weeks with volanesorsen 300 mg weekly.
- Outcomes assessed included changes in triglyceride and apoC-III levels, as well as risks of pancreatitis and adverse events.
- Both treatments demonstrated reductions in triglyceride and apoC-III levels over 26 and 52 weeks.
- No statistically significant differences were observed between olezarsen and volanesorsen for efficacy outcomes.
- Rates of acute pancreatitis and adverse events also were not significantly different.
- These results provide important comparative evidence to support clinical decision-making in FCS management.
- In the absence of statistically significant evidence of a difference in efficacy, treatment decisions should remain individualized and consider factors beyond comparative efficacy. Where clinically appropriate, switching to olezarsen may reduce patient burden due to its less frequent injection schedule and because it does not require platelet monitoring. Olezarsen’s generally mild and transient impacts on liver function test parameters may also be a relevant consideration for some patients.

## Supplementary Material


