# Supplementary material for: Comparative efficacy and safety of olezarsen versus volanesorsen for familial chylomicronemia syndrome: a matching-adjusted indirect comparison
Source: J Comp Eff Res. 2026 Jun 19;15(7):e260069. doi: 10.57264/cer-2026-0069 (PMC13329855; doi:10.57264/cer-2026-0069)
Supplement: Supplementary file 1 [file cer-15-260069-s1.docx]

## Appendix A: Systematic Literature Review

### PICO Criteria

**Table A1:** PICO eligibility criteria

| **PICOS item** | **Inclusion criteria** | **Exclusion criteria** |
| --- | --- | --- |
| **Population** | - Adult and pediatric patients with familial chylomicronemia syndrome (FCS) | - Secondary cause of hypertriglyceridemia (diabetes, hypothyroidism, obesity, alcohol consumption) - Animal studies, non-human studies (e.g., tissue, assay) |
| **Intervention** | - Olezarsen or plozasiran or volanesorsen | N/A |
| **Comparator** | - Any, placebo, or no treatment (i.e., no restrictions) | N/A |
| **Outcomes** | **Any Efficacy/Effectiveness outcomes** including but not limited to:   - Percentage change from baseline in fasting TG levels - Percentage change from baseline in fasting apo B-48 (apoB-48) and apoC-III - Percentage change from baseline in fasting HDL-C and non-HDL-C - Adjudicated acute pancreatitis event rate - Percentage of participants who achieve ≥ 40% or ≥ 70% reduction in fasting TG - Percentage of participants who achieve fasting TG ≤ 880 or ≤ 500 mg/dL   **Any Safety outcomes** including but not limited to:   - Adverse events (AEs) - Serious adverse events (SAEs) - Treatment-related AEs or SAEs - Withdrawal rates - Number of withdrawals due to any reason - Mortality - Treatment-related mortality | N/A |
| **Study design** | **Clinical trials**   - Randomized clinical trials - Non-randomized trials - Single arm trials - Pragmatic trials   **Observational Studies**   - Retrospective cohort studies - Prospective cohort studies - Cross-sectional studies - Case-control studies   **Included for library:**   - Systematic reviews, meta-analysis, ITC, MAIC* | - Review articles (e.g., narrative reviews) - Books, letters, notes, comments - Case reports/series - Pre-clinical studies |
| **Additional criteria (limits)** | | |
| **Timing** | None | |
| **Setting** | None | |
| **Language** | English | |
| **Publication date limit** | None | |

AE: adverse event; apoB: apolipoprotein B; apoC: apolipoprotein C; HDL-C: high-density lipoprotein cholesterol; ITC: indirect treatment comparison; MAIC: matching adjusted indirect treatment comparison; N/A: not applicable; SAE: serious adverse event

***Bibliographies of previously published SLR, meta-analysis, ITC, MAIC will be searched to identify relevant evidence.**

### Search Strategies for Main Electronic Databases

**Table A2:** Search strategy for Embase® via OvidSP

| **Database: Embase 1974 to 2024 August 01**  **Search executed: August 02, 2024** | | |
| --- | --- | --- |
| **#** | **String** | **Hits** |
| 1 | exp lipoprotein lipase deficiency/ | 1316 |
| 2 | exp hyperlipoproteinemia type 1/ | 1316 |
| 3 | Familial chylomicronemia.mp. | 432 |
| 4 | Familial hyperchylomicronemia.mp. | 61 |
| 5 | familial Hypertriglyceridemia.mp. | 226 |
| 6 | lipoprotein lipase deficiency.mp. | 485 |
| 7 | (hyperlipoproteinemia type 1 or hyperlipoproteinemia type I).mp. | 1333 |
| 8 | or/1-7 | 1831 |
| 9 | (exp animal/ or nonhuman/) not exp human/ | 7395417 |
| 10 | (Ephemera or "Introductory Journal Article" or News or "Newspaper Article" or Editorial or Comment or Overall or Letter or Short Survey or Tombstone or Books).pt. or in vitro study/ or (commentary or editorial or comment or letter or mice or rat or mouse or animal or murine).ti. | 5498569 |
| 11 | case reports.pt. or case report$.jw. or (case report or case study or case series or woman or man or child or adolescent or female or male or boy or girl or infant or unusual case).ti. | 1291201 |
| 12 | or/9-11 | 12238673 |
| 13 | (conference or conference abstract or conference review).pt. | 5990318 |
| 14 | limit 13 to yr="2022 -Current" | 749907 |
| 15 | 13 not 14 | 5240411 |
| 16 | 12 or 15 | 16569849 |
| 17 | 8 not 16 | **1196** |

**Table A3:** Search strategy for MEDLINE® via OvidSP

| **Database: MEDLINE ALL 1946 to August 01, 2024**  **Search executed: August 02, 2024** | | |
| --- | --- | --- |
| **#** | **String** | **Hits** |
| 1 | exp lipoprotein lipase deficiency/ | 669 |
| 2 | Familial chylomicronemia.mp. | 243 |
| 3 | Familial hyperchylomicronemia.mp. | 132 |
| 4 | familial Hypertriglyceridemia.mp. | 147 |
| 5 | lipoprotein lipase deficiency.mp. | 334 |
| 6 | (hyperlipoproteinemia type 1 or hyperlipoproteinemia type I).mp. | 683 |
| 7 | or/1-6 | 1088 |
| 8 | (exp animal/ or nonhuman/) not exp human/ | 5244920 |
| 9 | (Ephemera or "Introductory Journal Article" or News or "Newspaper Article" or Editorial or Comment or Overall or Letter or Short Survey or Tombstone or Books).pt. or in vitro study/ or (commentary or editorial or comment or letter or mice or rat or mouse or animal or murine).ti. | 3857162 |
| 10 | case reports.pt. or case report$.jw. or (case report or case study or case series or woman or man or child or adolescent or female or male or boy or girl or infant or unusual case).ti. | 3064349 |
| 11 | or/8-10 | 10712801 |
| 12 | 7 not 11 | **707** |

**Table A4:** Search strategy for Cochrane Central Register of Controlled Trials via OvidSP

| **Database: CENTRAL June 2024**  **Search executed: August 02, 2024** | | |
| --- | --- | --- |
| **#** | **String** | **Hits** |
| 1 | exp lipoprotein lipase deficiency/ | 29 |
| 2 | Familial chylomicronemia.mp. | 57 |
| 3 | familial Hypertriglyceridemia.mp. | 4 |
| 4 | lipoprotein lipase deficiency.mp. | 6 |
| 5 | (hyperlipoproteinemia type 1 or hyperlipoproteinemia type I).mp. | 63 |
| 6 | or/1-5 | 84 |
| 7 | (exp animal/ or nonhuman/) not exp human/ | 3665 |
| 8 | (Ephemera or "Introductory Journal Article" or News or "Newspaper Article" or Editorial or Comment or Overall or Letter or Short Survey or Tombstone or Books).pt. or in vitro study/ or (commentary or editorial or comment or letter or mice or rat or mouse or animal or murine).ti. | 24202 |
| 9 | case reports.pt. or case report$.jw. or (case report or case study or case series or woman or man or child or adolescent or female or male or boy or girl or infant or unusual case).ti. | 39148 |
| 10 | or/7-9 | 66526 |
| 11 | 6 not 10 | **83** |

### Search Strategies for Gray Literature

**Table A5:** Search strategy for US clinical trial registry (<https://clinicaltrials.gov/>)

| **Database: ClinicalTrials.gov**  **Date of search: August 19, 2024** | | |
| --- | --- | --- |
| **#** | **Search String** | **Hits** |
| 1 | Condition or Disease: Familial Chylomicronemia Syndrome  Study Results: Completed  Phase: All | 9 |

**Table A6:** Search strategy for EU clinical trial registry (<https://www.clinicaltrialsregister.eu/>)

| **Database: ClinicalTrialsRegister.eu**  **Date of search: August 19, 2024** | | |
| --- | --- | --- |
| **#** | **Search String** | **Hits** |
| 1 | Search Terms: Familial Chylomicronemia Syndrome  Results Status: Completed | 6 |

### PRISMA Flow Diagram

**Figure A1:** PRISMA flow diagram

Records identified from:

Embase (k = 1,196)

MEDLINE® (k = 707)

CENTRAL (k = 83)

Records removed *before screening*:

Duplicate records removed (k = 463)

Records screened

(k = 1,523)

Records excluded

(k = 1,501)

Records sought for retrieval

(k = 22)

Records excluded:

Outcomes (k = 1)

Study design (k = 6)

Duplicate (k = 1)

Other* (k = 7)

Records identified from:

Hand searching (k = 1)

Publications included in review

(k = 8) pertaining to 7 unique studies

**Screening**

Records sought for retrieval

(k = 1)

**Identification of studies via databases and registers**

**Identification of studies via other methods**

**Identification**

**Included**

Records assessed for eligibility (k = 1)

Records assessed for eligibility (k = 22)

*No additional outcomes than primary publication; k: number of studies

## Appendix B: Matching-Adjusted Indirect Comparison

### Distributions of patient weights

**Figure B1**: Histograms of patient weights for the risk of at least one AP episode, TEAE, and TRAE analyses.


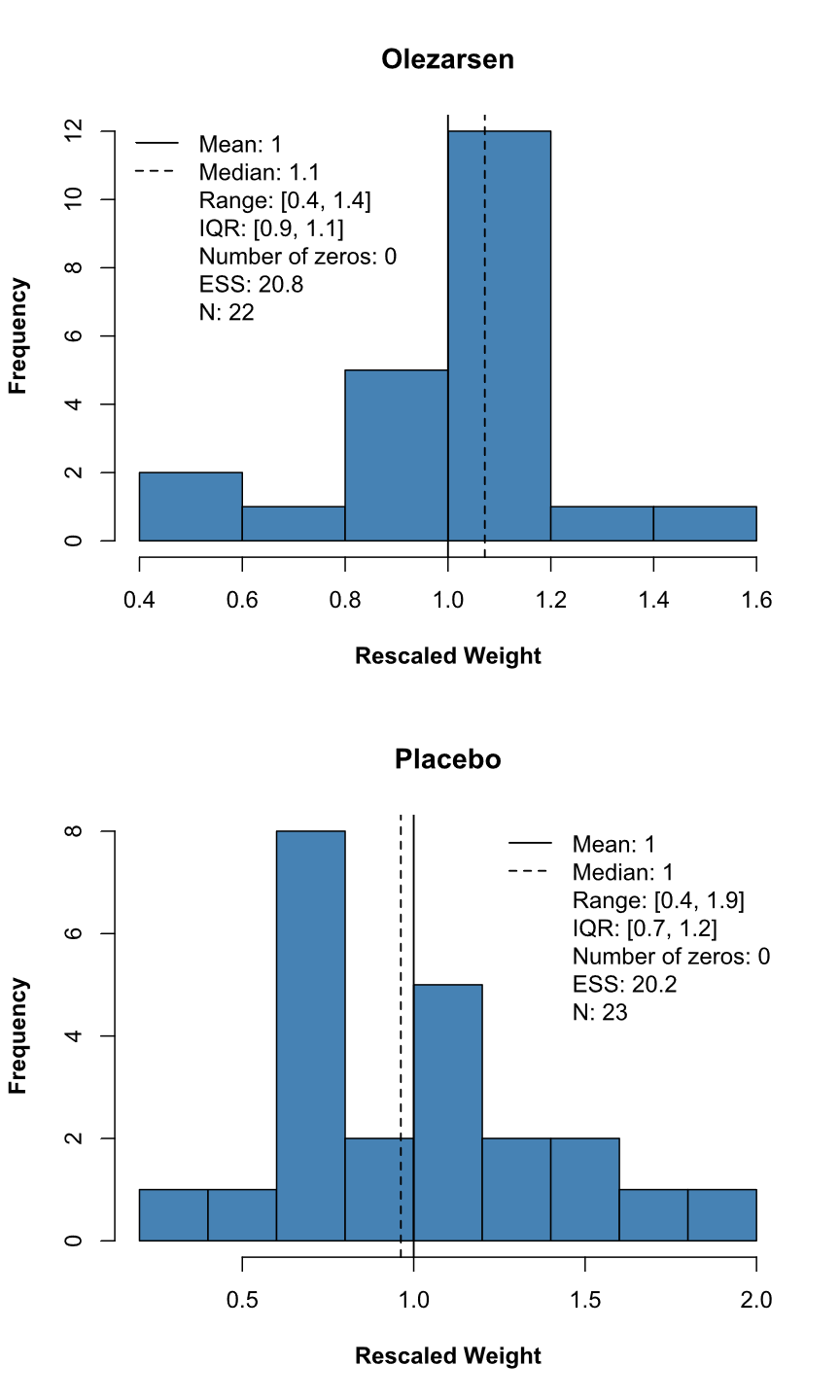


Abbreviations: AP – Acute Pancreatitis, ESS – Effective Sample Size, IQR – Interquartile Range, N – Sample size, TEAE – Treatment-Emergent Adverse Events, TRAE – Treatment-Related Adverse Events.

**Figure B2**: Histograms of patient weights for number of AP events analysis.


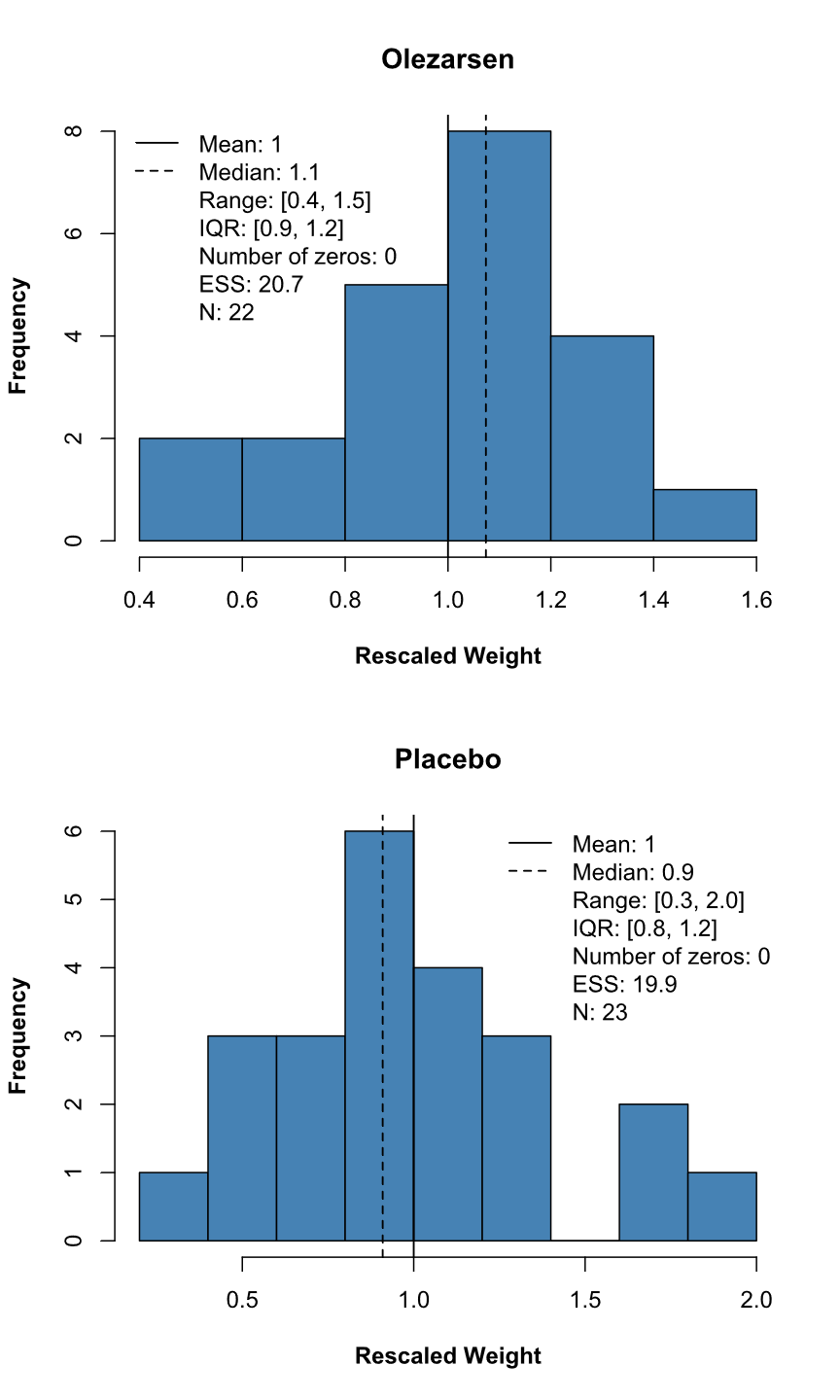


Abbreviations: AP – Acute Pancreatitis, ESS – Effective Sample Size, IQR – Interquartile Range, N – Sample size.

**Figure B3**: Histograms of patient weights for the fasting apoC-III and TG analyses.


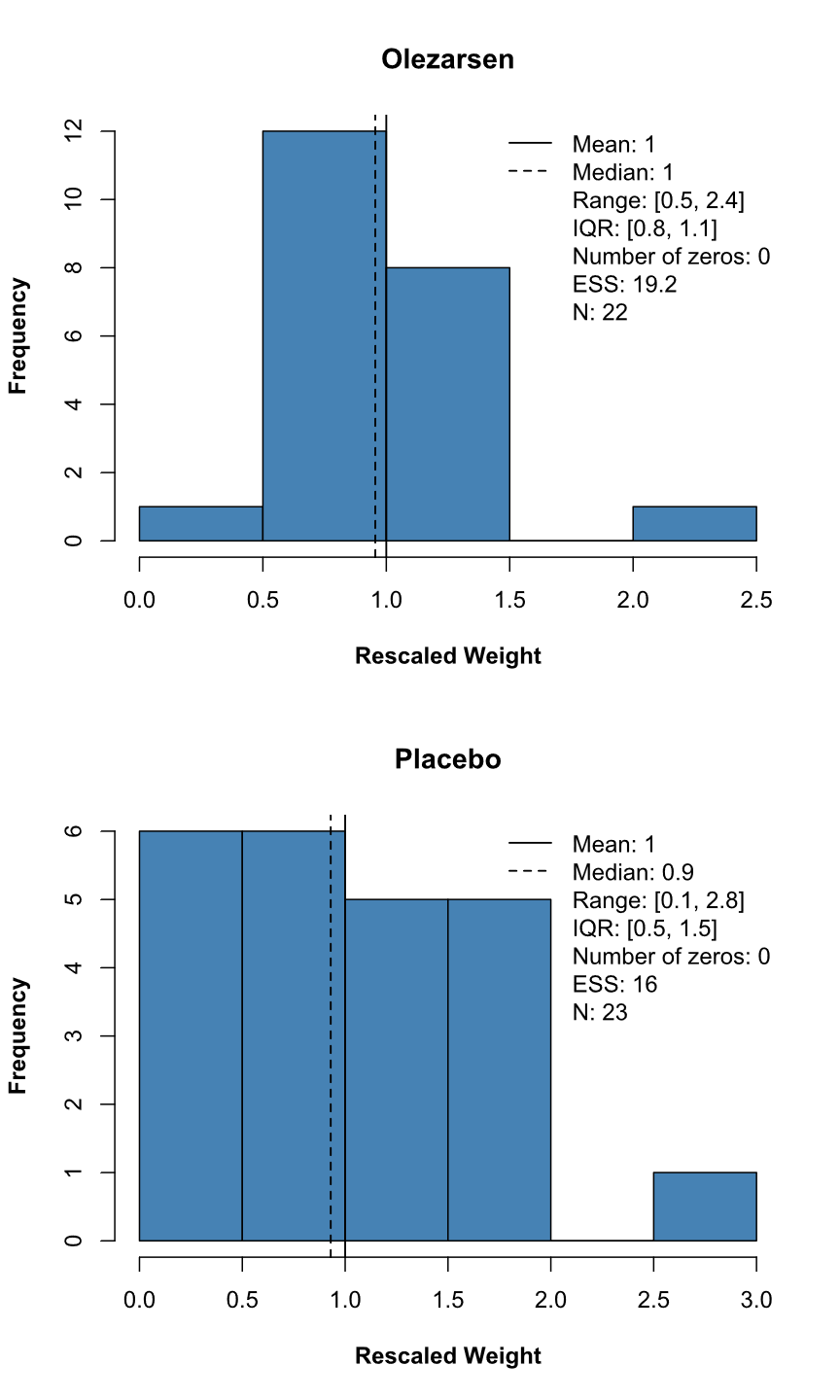


Abbreviations: apoC-III – Apolipoprotein C-III, ESS – Effective Sample Size, IQR – Interquartile Range, N – Sample size, TG - Triglycerides.

**Figure B4**: Histograms of patient weights for the SAE analysis.


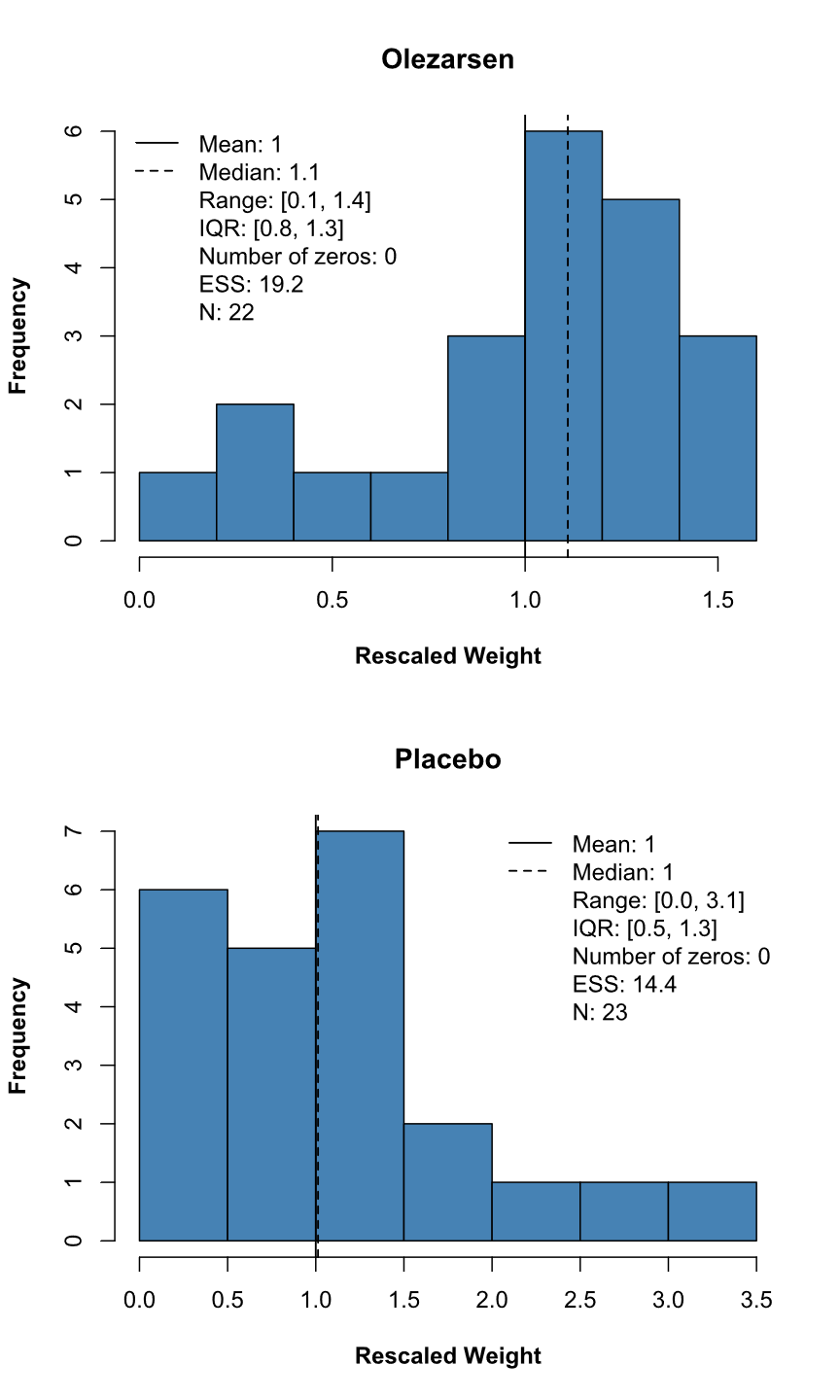


Abbreviations: ESS – Effective Sample Size, IQR – Interquartile Range, N – Sample size, SAE – Serious Adverse Event.
